# Beyond Gender Stereotypes in Language Comprehension: Self Sex-Role Descriptions Affect the Brain’s Potentials Associated with Agreement Processing

**DOI:** 10.3389/fpsyg.2015.01953

**Published:** 2015-12-23

**Authors:** Paolo Canal, Alan Garnham, Jane Oakhill

**Affiliations:** ^1^University of SussexBrighton, UK; ^2^Center for Neurocognition Epistemology and Theoretical Syntax, Institute for Advanced Study of PaviaPavia, Italy

**Keywords:** electrophysiology of language comprehension, individual differences, social perception of gender, P600, Nref, gender stereotypes, anaphor processing

## Abstract

We recorded Event-Related Potentials to investigate differences in the use of gender information during the processing of reflexive pronouns. Pronouns either matched the gender provided by role nouns (such as “king” or “engineer”) or did not. We compared two types of gender information, definitional information, which is semantic in nature (a mother is female), or stereotypical (a nurse is likely to be female). When they followed definitional role-nouns, gender-mismatching pronouns elicited a P600 effect reflecting a failure in the agreement process. When instead the gender violation occurred after stereotypical role-nouns the Event Related Potential response was biphasic, being positive in parietal electrodes and negative in anterior left electrodes. The use of a correlational approach showed that those participants with more “feminine” or “expressive” self sex-role descriptions showed a P600 response for stereotype violations, suggesting that they experienced the mismatch as an agreement violation; whereas less “expressive” participants showed an Nref effect, indicating more effort spent in linking the pronouns with the possible, although less likely, counter-stereotypical referent.

## Introduction

Research has shown that readers make inferences based on information that is explicit in a text, and on readily available general knowledge, to establish a coherent representation of the text. When a character is introduced in a text, readers use different sources of information to construct an incremental model of the discourse in which the representation of the character is specified to a greater or lesser extent. This representation creates expectations about what the character is likely to do or not to do. In the present study we explored the extent to which information that is not included in the text, and psychological factors that are unrelated to reading abilities, contribute to the representation of characters mentioned in the text. In particular we investigated how different types of information about gender, based either on the semantic definition of a noun or on stereotypical information associated with it, are used to determine the gendered representation of the text character. We also looked at whether a reader’s commitment to gender-related information is modulated by individual differences in the social perception of gender.

When reading a gender-specific noun (*mother* or *father*) or a proper name (*Alan* or *Jane*) the gender of the character is incorporated into the mental model of the discourse (Chang, 1980; Garnham and Oakhill, 1985). In a natural gender language such as English, in which nouns have no grammatical gender (although pronominal forms vary depending on the gender of their referents), gender information can be conveyed through definitional or stereotypical information (Corbett, 1991). Definitional gender derives from the semantic definition of a noun: *mothers* are women. Stereotypical gender by contrast is the gender bias that is often associated with “role” nouns such as *nurse*, which refer to professions and social roles, sometimes via titles. Stereotypical information about gender is not provided by grammar or semantics but derives at least partly from individuals’ world knowledge about the proportion of men and women carrying out certain jobs or holding certain social roles. Different studies (Kennison and Trofe, 2003; Gygax et al., 2008; Misersky et al., 2013) showed that the cognitive representation of a role name is often gender biased (e.g., *mechanics* are typically male). Therefore, from the simple mention of a *mechanic* people may infer that the noun’s referent is a man and strongly commit to this information (see the surgeon riddle in Sanford, 1985, p. 311; but also Banaji and Hardin, 1996; Carreiras et al., 1996; Osterhout et al., 1997; Sturt, 2003; Reynolds et al., 2006; Siyanova-Chanturia et al., 2012). Research has also shown that additional disambiguating information can interact with stereotype information, and override it (Carreiras et al., 1996; Duffy and Keir, 2004).

In the present study we used the Event Related Potential (ERP) technique, to compare how reading comprehension processes differ when people activate gender information that is categorical because it is semantic in nature (a female *mother*) and when they activate probabilistic information based on a stereotypical representation (a female *nurse*). ERPs are small voltage changes in the electrical activity of the brain, recorded from the scalp, consistently triggered by an external stimulus or a cognitive event. In comparison to the methodologies used in the majority of the studies cited above (reading times, response times, and eye-movements), knowledge about the functional meaning of ERP components, i.e., the neural activity generated by a neural source when a specific operation is performed (Luck, 2005), allows researchers to test hypotheses about qualitative differences in the processes under scrutiny.

As in Osterhout et al. (1997), a gender violation paradigm was used with reflexive pronouns as probes: a male or female character was introduced in a sentence and followed by a male or female pronoun. Pronouns explicitly signal that the entity to which they refer is female or male, so that reading *she* (or *herself*) rather than *he* (or *himself*), referring to *John* will result in some kind of cognitive cost, as previously reported in the psycholinguistic literature using behavioral (e.g., Caramazza et al., 1977) and ERP measures (e.g., Osterhout and Mobley, 1995). Pronouns are one instance of coreferential anaphoric expressions, i.e., words or phrases that refer to an entity previously introduced in the discourse (for an extensive overview of the mental processes involved in anaphor processing, see Garnham, 2001). An influential model of coreferential anaphor processing has been proposed by Garrod and Sanford (1994). Such processing involves at least two stages in which surface-form features and semantic-pragmatic factors interact in linking the anaphor to the appropriate referent, introduced by its antecedent. In the first stage (bonding), a loose attachment between the pronoun and potential antecedents is made on the basis of superficial information: this automatic process is constrained by lexical and syntactic factors. In the second stage (resolution), the link between pronoun and antecedent(s) made in the bonding process is evaluated and re-computed, if necessary taking into account the overall discourse representation. Both antecedent features (grammatical features, such as gender and number, but also accessibility of the antecedent, and discourse focus) and anaphor features (gender, number, and type) affect anaphor interpretation.

Many ERP studies have contributed to the identification of the cognitive mechanisms underlying anaphor processing (for a review see Callahan, 2008). The existing research investigates many different aspects of these mechanisms, ranging from referential ambiguity (Van Berkum et al., 1999, 2003, 2007; Nieuwland and Van Berkum, 2006), to the effects of processing repeated names (Camblin et al., 2007), from the role played by the antecedent’s features (Filik et al., 2008, 2011), to the direct comparison of different types of anaphor (Streb et al., 1999, 2004). The few ERP studies that provide evidence on the basic mechanisms of pronoun processing during sentence reading in English (Osterhout and Mobley, 1995; Osterhout et al., 1997), and thus are particularly relevant for the present study, found that when no antecedent is available for the anaphor, as in the sentence The aunt heard that *he, gender mismatching pronouns elicit an enhanced P600 component compared with gender-matching pronouns. The P600 component is a positive deflection observed in parietal electrodes, which develops in a late time-interval. Modulations of the P600 component were initially reported for morphosyntactic agreement manipulations (for a review, see Molinaro et al., 2011). Such effects are thought to represent difficulties in a late stage of processing, reflecting sentence revision or reanalysis processes (e.g., Kaan and Swaab, 2003; Friederici, 2011), they often involve syntactic information but more recently they have been observed during the processing of non-syntactic anomalies (e.g., Kuperberg, 2007; Brouwer et al., 2012).

The relation between anaphor and antecedent can be conceived of as semantic or “loose” agreement (e.g., Corbett, 1979) as the anaphor (target) has different forms depending on the referent’s (controller) semantic gender, but the domain in which referent and anaphor occur is often non-local (“unbound” personal pronouns can bind to antecedents outside the immediate clause containing them). In this study, we chose to focus on the processing of reflexive pronouns. Unlike definites, reflexives exhibit syntactically constrained behavior as they are governed by the verb, and their domain is local (Bosch, 1983; Principle A in Chomsky, 1993). When processing reflexive pronouns, rather than personal pronouns, readers should have strong expectations of finding a suitable antecedent, since reflexives must be coreferential, and thus they must agree with the antecedent in number and gender, otherwise the sentence would be syntactically anomalous. Indeed Osterhout et al. (1997) found that reading *The queen prepared himself* elicit an enhanced P600 response that is similar to what is observed in other cases of agreement violation (e.g., Molinaro et al., 2011).

But, what if a gender mismatch occurs on the basis of stereotypical gender information (e.g., *nurse* – *himself*)? As Osterhout et al. (1997) argued, one might expect that the anomaly of a male playing a stereotypically female role results from the evaluation of the pragmatic plausibility of the situation, and thus could be reflected in the modulation of the N400 component, which is associated with, among other things, the processing of semantically unexpected, or anomalous words (for review see Kutas and Federmeier, 2011). In contrast, Osterhout et al. (1997) found that stereotypical gender violations elicited a “syntactic” P600 effect, which was reduced in size, compared with the definitional gender case, but still reflected a qualitatively similar response to gender violation based on the noun’s semantics. This result is not necessarily surprising if we assume (i) that the activation of stereotypical gender information is the result of inference based on pragmatic knowledge that is carried out when processing the noun, but (ii) the use of this information (as with semantic gender information) can be controlled by syntactic factors when the pronoun explicitly requires evaluation of whether anaphor and antecedent are coreferential. Crucially, however, the two types of gender information differ: the gender of a *nurse* is not categorical as the gender of a *mother* is, but probabilistic. To process *he* referring to *nurse* when nurses are thought to be female in 74% of the cases (see British ratings in Misersky et al., 2013), should not be perceived as an outright agreement violation, as it would be if no possible referents were provided in the previous context. All that is necessary is to re-establish the appropriate, although less likely, reference to a male nurse. In the ERP literature on anaphor processing, the effort spent in establishing the appropriate reference when the antecedent is ambiguous, and thus difficult to link with the anaphor, has been associated with a frontal negativity dubbed the Nref effect: this negative deflection has been interpreted as reflecting the process of re-establishing the reference using information from the situation/discourse model (Van Berkum et al., 1999, 2003, 2007; Nieuwland and Van Berkum, 2006; Nieuwland, 2014).

Osterhout et al. (1997) carried out the first study using ERPs to compare the violation of gender expectations based on either semantic or stereotypical information. Their participants read sentences in which the gender of the introduced character could be semantically determined (*mother*, *king*) or stereotypically biased (*nurse*, *mechanic*). ERPs were time-locked to the presentation of reflexive pronouns that could either match the gender of the antecedent or not. The authors observed that the ERP response to both stereotypical and definitional gender violations affected the P600 component. This similarity was explained by postulating that stereotypical gender information is encoded in the grammar and thus produced “syntactic” P600 effects.

In the present study, as well as revisiting Osterhout et al.’s (1997) main results, we also use an individual differences approach. The rationale for using this approach derives from a specific result in Osterhout et al.’s (1997) study: definitional and stereotypical gender violation, but not subject-verb agreement violations, elicited larger P600 components for female participants than for male participants. One idea suggested by those authors referred to the possibility that “the amplitude of the positive shift reflects the ‘strength’ of stereotypic beliefs” (Osterhout et al., 1997, p. 281): to our knowledge this hypothesis has not been further tested and in the present study we will test it by exploring how differences in the social perception of gender are related to the way stereotype gender mismatch is processed. The variability in the P600 response between female and male participants suggests that considering mediating factors– instead of relying on average data and treating inter-individual variability as measurement noise – could provide a better understanding of the cognitive processes involved in a given mental operation (for a similar view, see Kanai and Rees, 2011).

In the present study we aimed to replicate Osterhout et al.’s (1997) and extend their findings by examining inter-individual variance and testing the hypothesis that the flexibility of the gender representation of a role noun might depend on the individual’s social perception of gender. A person who has strongly “sexist” attitudes might be less prone to accept a reference to a female *surgeon*, compared to a less “sexist” person. Or, if a person is more sensitive to gender stereotypes, she or he could activate gender information to a greater extent and thus show more difficulty in establishing the less likely reference. To test this hypothesis we looked for covariation between the electrophysiological effects associated with processing gender mismatching pronouns and individual scores on a battery of additional measures widely used in social psychology. These tests included both implicit and explicit measures and were designed to capture individuals’ perception of gender by monitoring the strength of the automatic associations between gender and career (Gender-Career Implicit Association Test – IAT, Greenwald et al., 1998), self sex-role descriptions (Bem Sex Role Inventory – BSRI, Bem, 1974), and explicit measures of sexism (Ambivalent Sexism Inventory – ASI, Glick and Fiske, 1996). Previous studies of individual differences in the ERP correlates of language processing have mostly used predictors that are specific to the language domain, such as (verbal) working memory (WM; e.g., Friederici et al., 1998; Vos and Friederici, 2003; Nieuwland and Van Berkum, 2006; Nieuwland, 2014), or, have considered the impact of proficency in monolingual native speakers (e.g., Pakulak and Neville, 2010), or individual differences in sentence processing for second language learners (e.g., Tanner et al., 2013; Tanner and Van Hell, 2014). The present work thus explored a more indirect link between non-domain-specific factors, such as social perception of gender, and the gendered representation of role-nouns and its effect on anaphor processing. We explored the impact of these variables using Linear Mixed Models (LMMs) on single trials. This is a relatively new and promising method for ERP research (e.g., Newman et al., 2012; Payne et al., 2015).

The experimental predictions are thus the following: in a minimal sentential context with only one available antecedent, the processing of a reflexive pronoun will incur processing costs if anaphor and antecedent do not match on gender. When the gender of the character is based on the noun’s semantics and is thus categorical, gender mismatch should elicit a P600 effect, because no appropriate referent is available. When the gender of the introduced character is instead based on a stereotypical representation, the link between anaphor and antecedent can in principle be made, if readers can mentally create a representation of a female mechanic. The establishment of a possible although less likely reference to a counter stereotypical representation might require additional inferential effort, and thus elicit an Nref effect. Furthermore, we expect to find individual variability in the response to mismatching pronouns in the stereotypical condition, and to capture some of this variability using the additional measures on the social perception of gender.

## Materials and Methods

### Ethics Statement

The experimental work reported in this paper was approved by the University of Sussex Life Sciences and Psychology Cross-Schools Research Ethics Committee. All procedures complied with the British Psychological Society’s Code of Human Research Ethics.

### Participants

Thirty-four right-handed native monolingual speakers of British English (17 female), with normal or corrected to normal vision, were recruited from the population of Sussex University to participate in the study. Ages ranged from 18 to 36 (mean = 20). Participants were paid £15 for their time. Three participants were removed from the final analyses because of excessive numbers of ERP artifacts.

### Additional Measures

After the ERP experiment, participants completed the battery of tests used to assess individual differences in the social perception of gender. Computerized versions of all the tests were used^1^ (programmed in PsyScope).

The gender-career Implicit Association Test (IAT; Greenwald et al., 1998) was presented following the guidelines from Greenwald’s website^2^ and the latest scoring algorithm was used (Greenwald et al., 2003). Briefly, in the Gender-Career IAT participants respond to a series of items from four categories: two represent the “concept discrimination”, i.e., men and women (five male and five female proper names) and two represent the “attribute discrimination”, i.e., career and family (seven career related words and seven family related words). Participants are asked to respond quickly by pressing one key for items representing one concept and one attribute (e.g., men and career in the related condition), and another key for items from the other two categories (e.g., women and family). Participants then perform the task again with the key assignment for one of the pairs switched (so that women and career share a response, and men and family). The IAT measure derives from the differences in response latencies between these two tasks (before and after the key assignment switch).

The Bem Sex-Role Inventory (BSRI; Bem, 1974) consists of a list of 60 words or phrases, and participants are asked to rate the degree to which they believe each word describes them, using a 7-point Likert scale. Twenty trials represent desirable masculine traits (e.g., “Acts as a leader”), 20 desirable feminine traits (e.g., “Affectionate”), and 20 neutral traits. From the BSRI three indexes are obtained: Androgyny (BEM), Masculinity (BEM-M), and Femininity (BEM-F). Masculinity and Femininity are the mean scores from the masculine and the feminine items, respectively. The Androgyny score is the absolute value of the Student t test ratio between masculinity and femininity scores (scores close to 0 thus indicate an androgynous person).

The Ambivalent Sexism Inventory (ASI; Glick and Fiske, 1996) consists of 22 statements about men and women and their relationships in contemporary society. Participants rate their agreement with the statements on a 6-point scale. The ASI is organized into two subscales measuring the constructs of Hostile Sexism (HS; e.g., She usually tries to put him on a tight leash.) and Benevolent Sexism (BS; e.g., Men should be willing to sacrifice their own well being in order to provide financially for the women in their lives.). The ASI (and BS and HS) scores are the mean scores, across items, on the scales.

### Stimuli

A set of 160 role nouns, including titles (e.g., *king*), states (e.g., *bachelor*), and occupations (e.g., *nurse*), was selected. The gender of half of the nouns was explicit and semantically defined (e.g., *mother*). In the other half, the gender was not explicit and could only be derived from the stereotype associated with the noun (e.g., *nurse*). The stereotypical gender of the nouns was taken from a previously collected database (Hamilton, 2006, unpublished data) in which people rated the role-nouns on an 11 point scale running from “strongly female” to “strongly male”. Participants were instructed to base their ratings on how the world is and not how it ought to be. We selected the 80 most male/female biased stereotypical role-nouns (40 female, 40 male) from the norms: the average rating of the nouns selected as stereotypically female was 3.21 (ranging from 1.63 to 4.79) whereas stereotypically male nouns obtained an average rating of 9.24 (ranging from 7.29 to 10.56).

One set of 160 sentences (plus 80 fillers) containing a noun in subject position and a reflexive pronoun as object of the main verb was created. In contrast to Osterhout et al. (1997), where more than 50% of the sentences had adjectives or other pre-nominal modifiers, the role nouns in the present study were not modified and were always followed by the main verb, to make sure that additional information would not further bias the gender representation of the nouns. Sentences continued for a few words following the reflexive pronouns (average 3.4 words). Two experimental lists were created using a latin-square design so that each participant was presented with each of the 160 role-nouns. Eighty sentences contained a definitionally male or female role-noun. In 40 of these sentences, the reflexive pronoun and subject agreed in number and gender, whereas in the other 40 sentences they disagreed. The other 80 target sentences contained a subject noun indicating a social role or occupation that was stereotypically male or female. The gender of the reflexive was consistent with the gender information provided by the role nouns in half of the sentences and inconsistent in the remainder (see **Table 1** for example sentences). Equal numbers (20) of male or female nouns were used in each condition. To keep the duration of the experiment below 75 min we restricted the number of filler sentences to 80, 40 of which were acceptable. Also to make the motivation of the experiment less obvious to our participants, 30 incorrect filler sentences contained pronoun-verb number agreement violations, instead of gender agreement anomalies. Ten semantic violations were then added to increase the variability in the materials. Hence, across all of the materials, 120 sentences were grammatically and semantically well formed and 120 were ill formed.

**Table 1:** Example of the experimental materials.

Type of noun	Agreement	Sentence	Condition
Definitional	Match	*The actress prepared herself to face the crowd.*	Definitional Match Condition
Definitional	Mismatch	*The actress prepared himself^∗^ to face the crowd.*	Definitional Mismatch Condition
Stereotypical	Match	*The architect saw himself in the mirror.*	Stereotypical Match Condition
Stereotypical	Mismatch	*The architect saw herself in the mirror.*	Stereotypical Mismatch Condition

### Procedure

Participants were tested individually in a dimly lit, sound attenuated room. They sat approximately 80 cm from a computer screen and were instructed to read the sentences carefully, as they would have to judge the acceptability of each sentence in terms of grammar and meaning. Each trial (presented in pseudo-randomized order) consisted of the following events: a fixation cross appeared at the center of the screen for 1000 ms, and was followed by word-by-word presentation of the sentence, with each word appearing for 350 ms at the center of the screen, followed by a 250 ms blank interval. Sentence final words were followed by a full stop. The acceptability question (“Was the sentence acceptable? Y or N”) appeared after a 1000 ms blank, which followed the final word of each sentence. Participants responded by pressing one of two buttons corresponding to yes/no answers (half of the participants responded “Y” with the left hand; the other half responded “Y” with the right hand). The question remained on screen until a response was given, after which the next trial began. Words were presented in white 18-point Arial font against a black background. Throughout the trial, appropriate triggers were sent to the EEG system, through the parallel port, using Presentation software^3^ The EEG session lasted for about 1 h, and the overall experimental session (EEG set-up, EEG recording, washing, and collection of the additional measures) lasted 120 min on average.

### EEG Recordings and Analysis

Electroencephalographic activity (EEG) was recorded from 35 Ag/AgCl electrodes (FP1, FP2, AF3, AF4, F7, F3, FZ, F4, F8, FT7, FC3, FCZ, FC4, FT8, T7, C3, CZ, C4, T8, CP5, CP1, CPZ, CP2, CP6, P7, P3, PZ, P4, P8, PO5, POZ, PO6, O1, OZ, O2) placed on the scalp using an elastic cap (Quik-Cap – Compumedics Neuroscan, Charlotte, NC, USA) following the Standard International 10–20 system. Vertical and horizontal eye movements were monitored with four electrodes, two placed beneath and above the left eye and two placed close to the left and right ocular canthi. Activity at the left and right mastoids (M1, M2) was also recorded. The EEG signal was referenced online to an electrode close to the vertex. Electrode impedance was kept below 5 kΩ at all scalp sites and mastoids, and below 15 kΩ for the eye electrodes. The EEG signal was amplified and digitized with a SynAmps2 amplifier (Compumedics Neuroscan, Charlotte, NC, USA) sampling at a rate of 250 Hz, and using a DC to 100 Hz low-pass filter during acquisition. The EEG signal was re-referenced oﬄine to the linked mastoids, and band-pass filtered from 0.05 to 45 Hz (second order Butterworth filter). The signal was then segmented in epochs from -350 to 1100 ms around the presentations of pronouns. In this time interval, artifact rejection was carried out determining an allowed maximum voltage range of 100 μV in each epoch, and through the visual inspection of the remaining epochs. Epochs from -150 to 1100 ms relative to critical word onset were selected for ERP analysis. The artifact-free epochs were baseline corrected by subtracting the mean amplitude in the 150 ms pre-stimulus interval from the post stimulus activity. Data processing was carried out using the EEGLAB (Delorme and Makeig, 2004) and FieldTrip (Oostenveld et al., 2010) open-source toolboxes for MATLAB (MathWorks, Natick, MA, USA). Thirty-one participants were included in the analysis with an average epoch loss of 13.46%. The total rejection rate for these participants ranged from 4.37 to 28.12% of the epochs.

We performed statistical analyses^4^ (using the R statistical package) in one time-window corresponding to the P600 canonical time-window, ranging from 500 to 900 ms. We used LMMs (lme4 package, Bates et al., 2015b) to account for the effects of within subjects factors and their interactions with the continuous covariates. LMMs lend themselves to ERP data (e.g., Bagiella et al., 2000; Newman et al., 2012) as they deal with non-sphericity, unbalanced experimental cells and, unlike ANCOVA, do not assume homogeneity of regression slopes across combinations of the independent variables. LMMs were used to predict the average ERP amplitude in the time window of interest for each epoch recorded during the experiment, except those excluded by the artifact rejection procedure, and “outliers” lying outside a fixed threshold of minimum and maximum allowed amplitude (+/-25 μV, 1.07% data loss) as the tails of the distribution departed from normality. Matrix size: 33 channels by 160 sentences by 31 participants.

Channels (except FP1 and FP2 as usuallly noisier than the rest of channels because placed close to the eye and front muscles) were organized by two topographic factors [Mediality: Left (all 12 left channels), Midline (all seven midline channels), Right (all 12 right channels); Longitude: Frontal (AF, F, and FC electrodes – 12 channels), Central (all C and CP electrodes – 10 channels), Parietal (all P, PO, and O electrodes – 11 channels)]. LMMs evaluated the effect of four within-subjects predictors (Agreement, Type of Noun, Longitude, and Mediality) and their interactions. Also the individual difference scores (after centering values on the mean of each covariate), and participants’ sex (in interaction with the covariates), entered the model as fixed effects. To warrant the conservativeness of the analysis we tested a model with maximal random structure as suggested by Barr et al. (2013). However, the high number of parameters (81) that the optimizer had to estimate determined a lack of convergence, which could be reached only when models had to estimate less than 25 parameters. Therefore, the number of factors in the random effects structure was determined on the grounds of feasibility (e.g., Bates et al., 2015a). The decision about which random slopes had to be included in the random structure was also constrained by feasibility (three levels factors – Longitude – easily increased the model complexity, compared to two levels factors) and by the fact that by subject and item random slopes for Agreement or Type of Noun should be included in the random structure to provide more conservative estimation of the factors that were manipulated. Since “random slopes for subjects pertain to properties of the words, and the random slopes for word pertain to properties of the subjects” (Baayen and Milin, 2010, p. 21) we further allowed by-item random slopes of two variables (Sex and BSRI-f). The reliability of the fixed effects was evaluated by model comparison using the LMERConfenienceFunctions package (Tremblay and Ransijn, 2015), as in Newman et al. (2012). In particular, a backfitting procedure was used, which compared models of decreasing complexity using log-likelihood ratio tests. The procedure removed terms in the model that did not make significant contribution to fit, to obtain a parsimonious model. To obtain a good compromise between computation time and conservativeness, we first backfitted the fixed effect structure on a simple random structure, and then we forward fitted the more complex random structure, including Sex and BSRI-f as the two individual factors that resulted the most significant fixed effects. Analysis of variance for each fixed effect is reported (F ratios between sum of squares of the model’s terms and the model’s residuals from the REML estimation), and lower-bound p values were calculated using the denominator degrees of freedom obtained by subtracting the number of estimated parameters from the number of data points, although the determination of the appropriate denominator degrees of freedom for such tests is at least problematic (e.g., Baayen et al., 2008). Main effects of topographic factors or interactions not involving the experimental factors (e.g., Mediality X Longitude or Longitude X Sex) are not reported as they can be considered irrelevant. Deviance coding was used for all categorical factors.

## Results

### Acceptability Judgments

Participants judged sentences as acceptable as follows: gender match and mismatch to semantically defined nouns, 92.10% (*SD* = 8.41%) and 16.58% (*SD* = 9.62%); gender match and mismatch to stereotypical gender nouns, 94.43% (*SD* = 7.26%) and 89.28% (*SD* = 12.78%). To evaluate the differences in acceptability judgments we used generalized mixed-models, using a binomial distribution. The model was specified as following: Agreement and Type of Noun were treated as fixed effects, whereas the random structure was maximally specified with by-subjects random intercepts and random slopes for Agreement by Type of noun and by-item random intercepts and random slopes for Agreement only, because the manipulation of type of noun was between-items. Reliable differences emerged between gender matching and mismatching pronouns in both Definitional (β = -4.51, *z* = -18.73, *p* < 0.001) and Stereotypical conditions (β = -0.70, *z* = -3.17, *p* < 0.01), although mismatching pronouns following stereotypical role nouns are far more acceptable than mismatching pronouns in the definitional condition (β = 4.40, *z* = 15.23, *p* < 0.001).

### Individual Differences

In **Table 2** the correlations between predictors from the battery of tests (BSRI, BSRI-m, BSRI-f, ASI, ASI-h, ASI-b, IAT) are reported. High correlations emerged between the scores obtained in subscales and global scores, for different tests: BSRI was correlated with the associated BSRI-m [*r* = -0.59, *t*(29) = -3.89, *p* < 0.001] and BSRI-f [*r* = 0.67, *t*(29) = 4.93, *p* < 0.001] subscales; ASI was correlated with ASI Hostile [*r* = 0.85, *t*(29) = 8.78, *p* < 0.001] and ASI Benevolent [*r* = 0.81, *t*(29) = 7.54, *p* < 0.001]. These correlations reflect collinearity between the main indexes and the subscales from which they are derived and, therefore, only BSRI and ASI subscales were further tested as predictors. Interestingly, a strong negative correlation between IAT and BSRI-m emerged [*r* = -0.68, *t*(29) = -5.00, *p* < 0.001]. We also tested by means of Welch two-sample *t*-tests whether male and female participants obtained significantly different scores on each scale: marginally significant differences due to participants sex emerged for ASI Hostile [men scored an average of 2.15 vs. an average of 1.65 for women, *t*(29.35) = 1.73, *p* < 0.1], whereas for the remaining scales no differences due to participants’ sex emerged [all *t*s < 1].

**Table 2:** Correlations between the seven measures derived from the battery of tests investigating social perception of gender.

Measure	1	2	3	4	5	6	7
(1) IAT	-						
(2) BSRI	0.49^∗^	-					
(3) BSRI-M	0.68^∗∗∗^	-0.59^∗∗^	-				
(4) BSRI-F	-0.03	0.67^∗∗∗^	0.16	-			
(5) ASI	0.07	-0.06	0.09	0.05	-		
(6) ASI-H	0.04	-0.19	0.02	-0.21	0.85^∗∗∗^	-	
(7) ASI-B	0.16	0.14	0.11	0.31ˆ	0.81^∗∗∗^	0.41^∗^	-

*Levels of significance are indicated by ∧, <0.1; ^∗^, <0.05; ^∗∗^, <0.01; ^∗∗∗^, <0.001.*

### Event related Potentials

**FIGURE 1:**
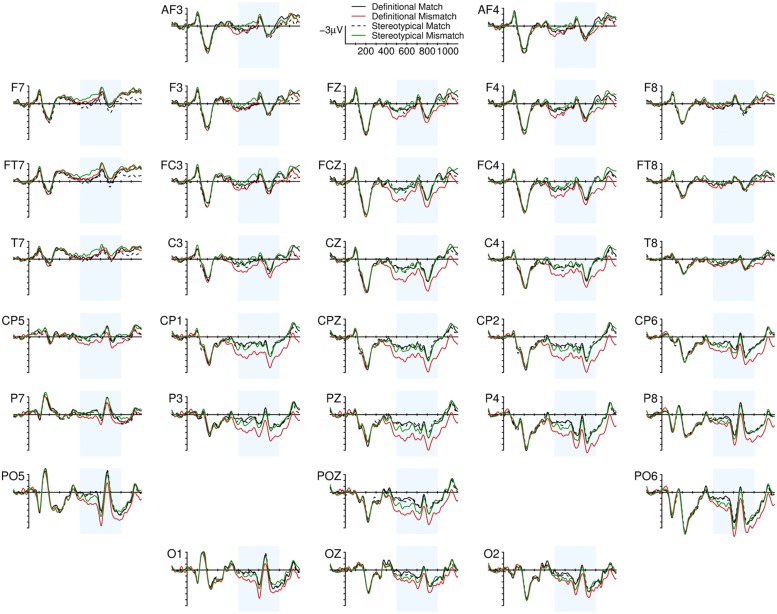
**Grand Average Event Related Potential (ERP) response time-locked to the visual presentation of reflexive pronouns.** The ERP recorded from 33 electrodes associated with Definitional Match condition (black solid line), Definitional Mismatch condition (red solid line), Stereotypical Match condition (black dashed line), Stereotypical Mismatch condition (green solid line) are displayed. Negative polarity is plotted upward.

**FIGURE 2:**
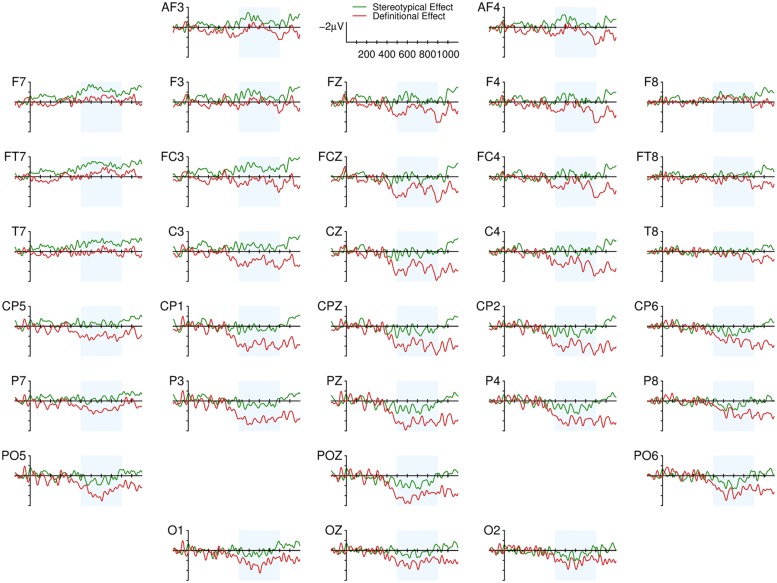
**Difference Waves (Mismatch minus Match) in Stereotypical and Definitional conditions.** Difference waves were obtained by subtracting ERPs to pronouns that agreed with the gender of Stereotypical (green line) or Definitional (red line) role-nouns from those that disagreed. Negative polarity is plotted upward.

From a visual inspection of the grand averages (**Figure 1**), time-locked to the presentation of the reflexive pronoun, the effect of gender mismatch is evident in the Definitional condition. Its broad and posterior distribution, its timing (450 ms to the end of the epoch) and the polarity of the effect are compatible with a modulation of the P600 component. In the Stereotypical condition, the effect of mismatch is less clear: there seems to be a positive deflection in posterior and right lateralized electrodes in a narrower time window (500–750 ms) that is consistent with a P600 effect. Moreover, gender mismatching pronouns also elicit a negative deflection in frontal left electrodes which temporally overlaps to the parietal Positivity in the 500 to 900 ms time-window (see also **Figure 2**). Looking at the grand averages and the difference waves, we fitted models with the following contrasts on the topographic factors. Because of the left frontal negative deflection for stereotype mismatching pronouns we coded the Mediality factor using Left as the reference level for comparisons with the Mediality and Right levels. The Parietal level of the Longitude factor was the reference for comparisons with Frontal and Central.

**FIGURE 3:**
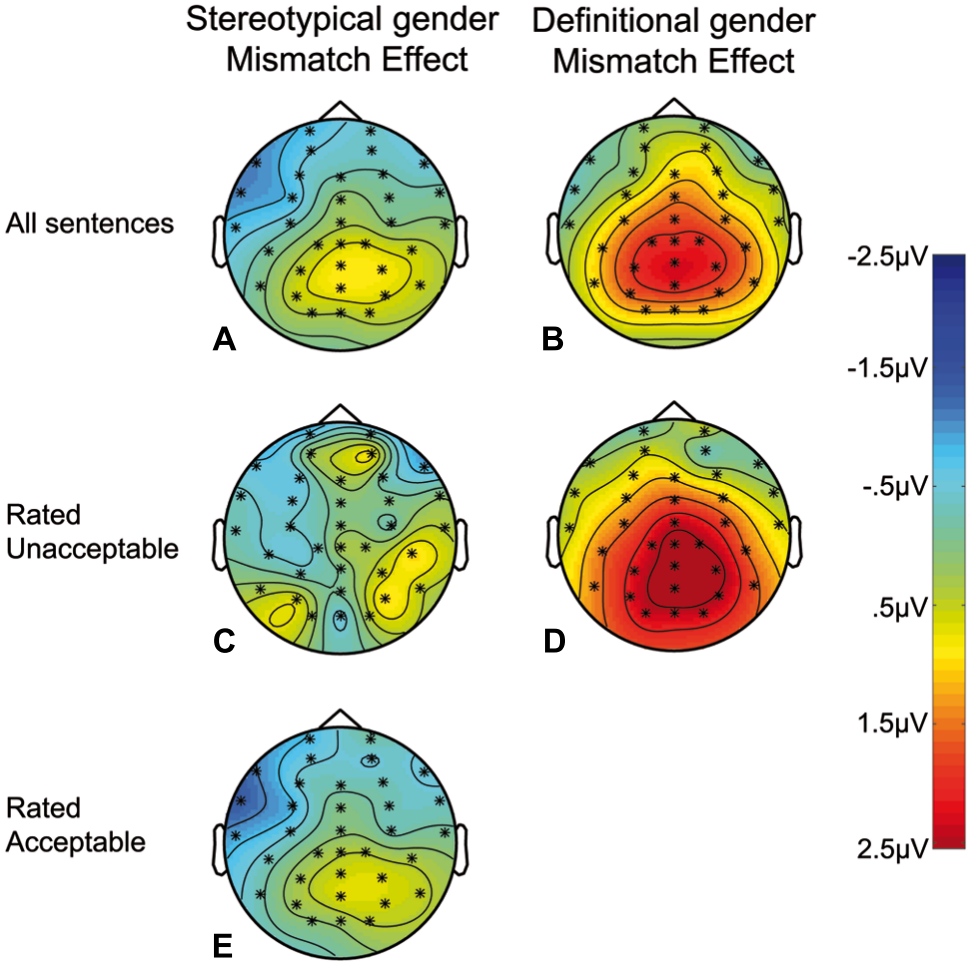
**Scalpmaps representing the distribution of agreement effect (Mismatch minus Match) in Stereotypical and Definitional conditions.** The ERP differences (from 500 to 900 ms) of two types of contrasts is shown: **(A,B)** represent Stereotypical and Definitional mismatch effects, respectively. **(C,D)** represent the distribution of the effect obtained by subtracting the ERPs to reflexives in sentences that were rated as “acceptable” from those in sentences rated as “unacceptable”, in the Stereotypical and Definitional conditions, respectively. The bottom scalp-map **(E)** represents ERP differences between Stereotypical gender mismatching and Stereotypical gender matching sentences for all sentences that were rated as “acceptable”.

Statistical analysis carried out in the P600 time window (500–900 ms) confirmed that agreement affects pronoun processing in the Stereotypical and Definitional conditions to a different extent [Agreement × Type of Noun: *F*(1,141489) = 175.62, *p* < 0.001]. The mismatch effect is larger in the Definitional condition [*M* = +0.80 μV] compared to the Stereotypical condition [*M* = -0.02 μV]. The effect of Agreement on the ERPs is focussed on more posterior locations [Agreement × Longitude *F*(2,141489) = 56.12, *p* < 0.001], and this pattern is consistent with the canonical distribution of the P600 component as being larger in Parietal with respect to Frontal electrodes [*M*_Frontal_ = -0.13 μV vs. *M*_Parietal_ = +0.80 μV, *t* = 10.15] and Central electrodes [*M* = +0.33 μV vs. *M*_Central_, *t* = 3.34]. However, results show also that the effect has an asymmetric distribution [Agreement × Longitude × Mediality *F*(4,141489) = 3.59, *p* < 0.01]. This complex interaction (**Figure 3**; **Table 3**) reflects the fact that the effect was not different between levels of Mediality (*M*_LeftvsMidline_ = -0.16 μV, *M*_LeftvsRight_ = -0.27 μV, *M*_CentralvsRight_ = +0.09 μV) in Parietal electrodes, it was focused along the midline on central electrodes (*M*_LeftvsMidline_ = -0.96 μV, *M*_LeftvsRight_ = -0.52 μV, *M*_CentralvsRight_ = +0.43 μV), and was reduced in Frontal (*M*_LeftvsMidline_ = -0.76 μV, M_LeftvsRight_ = -0.62 μV, *M*_CentralvsRight_ = +0.14 μV) – and reversed in left Frontal – electrodes (**Figures 3A,B**).

**Table 3:** **Definitional condition.** ANOVA table for the Event Related Potential (ERP) amplitude during the time window of interest (500–900 ms).

Factor	df	SumSq	MeanSq	*F*	dendf	pval	Sig
Agreement	1	558.11	558.11	10.79	70412	0.001	^∗∗^
Agreement:Longitude	2	3162.96	1581.48	30.58	70412	0	^∗∗∗^
Agreement:Mediality	2	1202.89	601.44	11.63	70412	0	^∗∗∗^
Agreement:BSRI-f	1	1.03	1.03	0.02	70412	0.8878	
Agreement:BSRI-m	1	130.71	130.71	2.53	70412	0.1119	
Agreement:ASI-b	1	5.32	5.32	0.10	70412	0.7483	
Agreement:ASI-h	1	3.61	3.61	0.07	70412	0.7916	
Agreement:Sex	1	3.41	3.41	0.07	70412	0.7972	
Agreement:Longitude:Mediality	4	480.70	120.18	2.32	70412	0.0542	
Agreement:Longitude:BSRI-f	2	866.78	433.39	8.38	70412	0.0002	^∗∗∗^
Agreement:Longitude:IAT	2	133.20	44.40	0.86	70412	0.4618	
Agreement:Longitude:ASI-b	2	1110.81	555.40	10.74	70412	0	^∗∗∗^
Agreement:Longitude:ASI-h	2	218.81	109.40	2.12	70412	0.1206	
Agreement:Sex:BSRI-f	1	1.43	1.43	0.03	70412	0.8681	
Agreement:Sex:BSRI-m	1	157.33	157.33	3.04	70412	0.0812	
Agreement:Sex:IAT	1	725.70	725.70	14.03	70412	0.0002	^∗∗∗^
Agreement:Mediality:IAT	2	286.68	143.34	2.77	70412	0.0626	
Agreement:Mediality:ASI-b	2	253.94	126.97	2.45	70412	0.0859	
Agreement:Mediality:ASI-h	2	320.01	160.01	3.09	70412	0.0453	^∧^
Agreement:Longitude:Sex:BSRI-f	2	1597.59	798.80	15.44	70412	0	^∗∗∗^
Agreement:Longitude:Sex:BSRI-m	2	500.81	125.20	2.42	70412	0.0461	^∧^
Agreement:Mediality:Sex:BSRI-m	2	473.45	118.36	2.29	70412	0.0574	
Agreement:Mediality:Sex:IAT	2	286.98	143.49	2.77	70412	0.0624	

Levels of significance are indicated by ^∧^, <0.05; *, <0.025; **, <0.005; ***, <0.0005.

Concerning the effect of participant sex and that of individual covariates, several significant three-way (nine) and four-way (eight, of which six involved participant Sex) interactions emerged. Also one five-way interaction was significant [Agreement × Noun × Longitude × Sex × BSRI-f: *F*(2,141489) = 10.37, *p* < 0.001]. It is worth noting that the Agreement × Noun Type interaction was further modulated by individual differences in BSRI-f [*F*(1,141489) = 93.10, *p* < 0.001], BSRI-m [*F*(1,141489) = 48.51, *p* < 0.001] and ASI-h [*F*(1,141489) = 8.77, *p* < 0.01] and also by an Agreement × Noun Type × Longitude × ASI-h interaction [*F*(2,141489) = 15.53, *p* < 0.001], but not by Sex [*F* < 1]: these interactions always have the same pattern representing a stronger modulation of the ERP effect by individual differences in the Stereotypical condition, than in the Definitional condition. To better describe this pattern of results we broke down the analysis by running two subsidiary models, on Definitional and Stereotypical role-nouns data, separately.

### LMM Results on Defintional Role-Nouns

A main effect of Agreement [*F*(1,70412) = 10.79, *p* < 0.01] emerged. It was modulated by Longitude [*F*(2,70412) = 30.58, *p* < 0.001] and Mediality [*F*(2,70412) = 11.63, *p* < 0.001]. Planned contrasts confirmed the posterior distribution of the effect: differences between mismatching and matching pronouns in Parietal (*M* = +1.23 μV) compared to Frontal (*M* = +0.25 μV) electrodes were in fact consistent (*t* = +7.02), whereas the effect in Central electrodes (*M* = +1.00 μV) was less pronounced with respect to that recorded in Parietal electrodes (*t* = -3.19). The interaction between Agreement and Mediality revealed reliable differences in the effect of mismatch between Left (*M* = +0.51 μV) and Midline (*M* = +1.11 μV) electrodes (*t* = -4.52), and less pronounced differences between Left and Right (*M* = +0.87 μV) electrodes (*t* = -4.04), supporting the idea that the mismatch effect was more focused on Midline electrodes, and particularly reduced in Frontal and Left scalp sites (with a marginally significant Agreement × Longitude × Mediality interaction).

**FIGURE 4:**
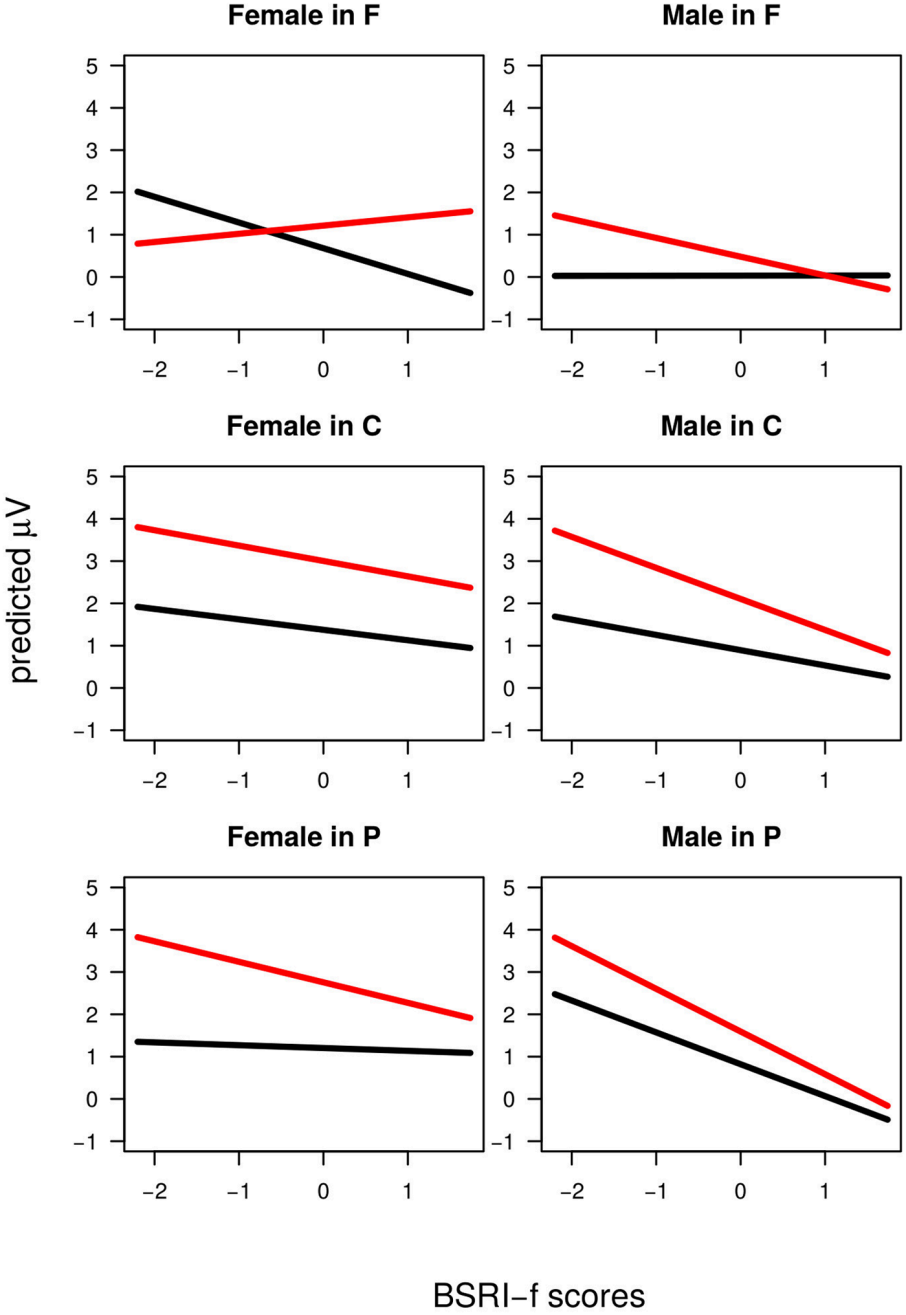
**Definitional condition.** The Agreement × Longitude × Sex × BSRI-f interaction. The *x*-axis is BSRI-f scores, and the *y*-axis is amplitude. The solid black line is the Agreement Match condition and the solid red line is the Agreement Mismatch condition. Top row represents electrodes in Frontal scalp-locations, middle row represents Central scalp-locations and bottom row represents EEG in Parietal scalp-locations. In the left column results for female participants are displayed. In the right column results for male participants are displayed.

**FIGURE 5:**
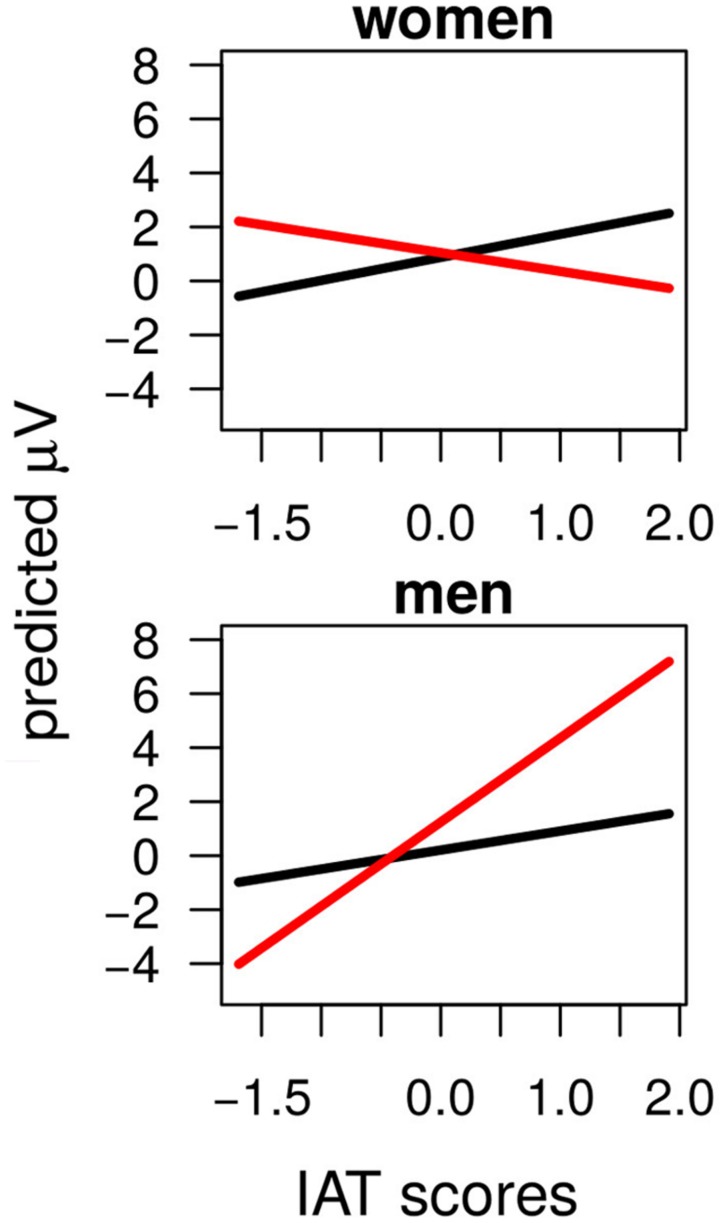
**Definitional condition.** The Agreement × Sex × IAT interaction. The *x*-axis is IAT scores, and the *y*-axis is amplitude. The solid black line is the Agreement Match condition and the solid red line is the Agreement Mismatch condition. The top figure shows results for female participants from all scalp-locations. The bottom figure shows results for male participants from all scalp-locations.

Notably, when considering the effect of individual covariates different significant interactions emerged involving BSRI-f, ASI-b, Sex, and IAT (**Table 4**). ASI-b and BSRI-f were involved in similar interactions with Longitude and Agreement. Probably because of modearate collinearity [*r* = 0.31, *t*(30) = 1.81, *p* < 0.1] between these two measures, even though the *F* values for both interactions were large, the change in slope between Agreement conditions across levels of Longitude, did not consistently vary with ASI-b scores [Frontal vs. Parietal β_diff_ = -0.13, *t* < 1; Central vs. Parietal β_diff_ = -0.03, *t* < 1] but did so with BSRI-f scores [Frontal vs. Parietal β_diff_ = +0.48, *t* = 3.81; Central vs. Parietal β_diff_ = +0.07, *t* < 1]. Indeed, the effect of BSRI-f had a stronger impact in the EEG value as it was further qualified by the Agreement × Longitude × Sex × BSRI-f interaction which attested to differences between male and female participants in the BSRI-f modulation of the Agreement effect (**Figure 4**): such differences were strong comparing BSRI-f slope change associated with the Agreement effect between Male and Female participants in Frontal – where women showed β = +0.72 and men β = -0.45 – vs. Parietal – where women showed β = -0.38 and men β = -0.25 – electrodes. Such differences were reliable in the comparison between Frontal and Parietal electrodes [β = +1.36, *t* = 4.87] but not in the comparison between Central and Parietal electrodes [β = +0.39, *t* = 1.34]. The ERP pattern as modulated by BSRI-f was thus similar for Male and Female participants in Central and Parietal electrodes (as also showed by the Agreement by Longitude by BSRI-f significant interaction). However, in Frontal electrodes the pattern was inversed. Female participants showed larger Frontal Positivity associated with an increase in BSRI-f scores, whereas Male participants showed a reduction of the Frontal portion of the P600 associated with an increase in BSRI-f scores. Moreover, participants Sex was involved in a Agreement × Sex × IAT interaction (**Figure 5**): the size of the Mismatch effect (across all scalp-sites) increased as function of IAT score (β = +2.23) for male participants and decreased (β = -1.21) for female participants (β_diff_ = +3.44, *t* = 3.70).

**Table 4:** **Sterotypical condition.** ANOVA table for the Event Related Potential (ERP) amplitude during the time window of interest (500–900 ms).

Factor	df	SumSq	MeanSq	*F*	dendf	pval	Sig
Agreement	1	15.36	15.36	0.30	71038	0.5859	
Agreement:Longitude	2	2678.87	1339.44	25.87	71038	0	^∗∗∗^
Agreement:Mediality	2	1385.03	692.51	13.38	71038	0	^∗∗∗^
Agreement:BSRI-f	1	496.52	496.52	9.59	71038	0.002	^∗∗^
Agreement:BSRI-m	1	6.62	6.62	0.13	71038	0.7206	
Agreement:ASI-b	1	9.68	9.68	0.19	71038	0.6655	
Agreement:ASI-h	1	40.69	40.69	0.79	71038	0.3753	
Agreement:Sex	1	13.51	13.51	0.26	71038	0.6094	
Agreement:Longitude:Mediality	4	289.66	72.41	1.40	71038	0.2315	
Agreement:Longitude:BSRI-f	2	120.76	60.38	1.17	71038	0.3116	
Agreement:Longitude:IAT	2	178.64	59.55	1.15	71038	0.3272	
Agreement:Longitude:ASI-b	2	15.71	7.85	0.15	71038	0.8592	
Agreement:Longitude:ASI-h	2	1590.88	795.44	15.36	71038	0	^∗∗∗^
Agreement:Sex:BSRI-f	1	95.22	95.22	1.84	71038	0.1751	
Agreement:Sex:BSRI-m	1	0.26	0.26	0.01	71038	0.9436	
Agreement:Sex:IAT	1	32.78	32.78	0.63	71038	0.4262	
Agreement:Mediality:IAT	2	376.47	188.24	3.64	71038	0.0264	^∧^
Agreement:Mediality:ASI-b	2	6.25	3.12	0.06	71038	0.9414	
Agreement:Mediality:ASI-h	2	112.48	56.24	1.09	71038	0.3375	
Agreement:Longitude:Sex:BSRI-f	2	84.47	42.24	0.82	71038	0.4423	
Agreement:Longitude:Sex:BSRI-m	2	114.78	28.69	0.55	71038	0.6959	
Agreement:Mediality:Sex:BSRI-m	2	389.27	97.32	1.88	71038	0.1109	
Agreement:Mediality:Sex:IAT	2	93.47	46.73	0.90	71038	0.4055	

Levels of significance are indicated by ^∧^, <0.05; *, <0.025; **, <0.005; ***, <0.0005.

### LMM Results on Stereotypical Role-Nouns

The effect of Agreement was modulated by Longitude [*F*(2,70038) = 25.86, *p* < 0.001] and by Mediality [*F*(2,70038) = 13.38, *p* < 0.001]. The mismatch effect differed [+0.92 μV, *t* = 7.20] between Frontal (*M* = -0.54 μV) and Parietal electrodes (*M* = +0.38 μV) and also between Parietal and Central (*M* = +0.05 μV) electrodes [+0.44 μV, *t* = 3.19]. Electrodes over the Left hemisphere (*M* = -0.48 μV) showed a different gender mismatch effect from both Midline (*M* = +0.17 μV, *t* = -4.95) and Right (*M* = +0.09 μV, *t* = -4.05) lateralized electrodes. These results confirm that that the gender mismatch effect in the stereotypical condition is associated with a Frontal, and Left negativity overlapping with a Parietal positivity.

**FIGURE 6:**
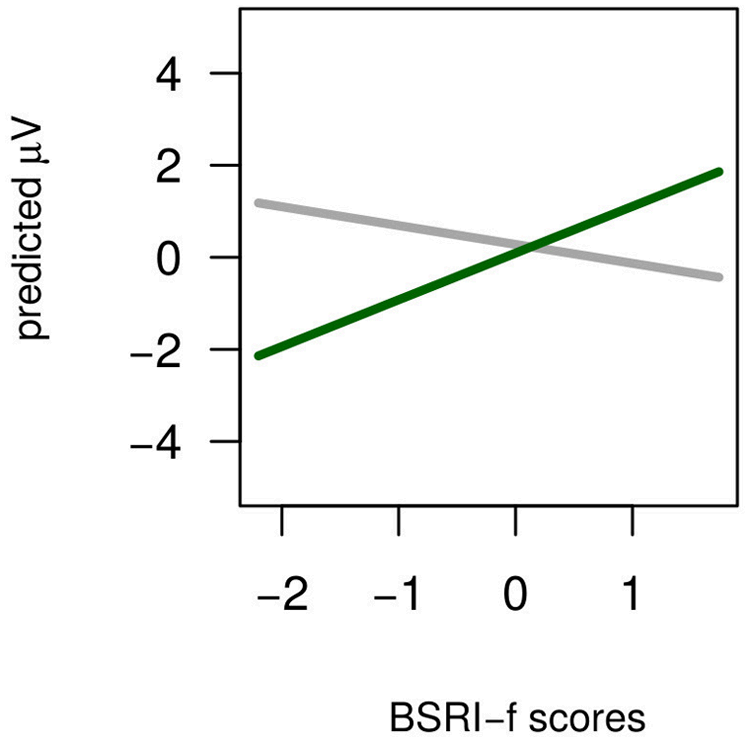
**Stereotypical condition.** The Agreement × BSRI-f interaction. The *x*-axis is BSRI-f scores, and the *y*-axis is amplitude. The solid gray line is the Agreement Match condition and the solid green line is the Agreement Mismatch condition.

**FIGURE 7:**
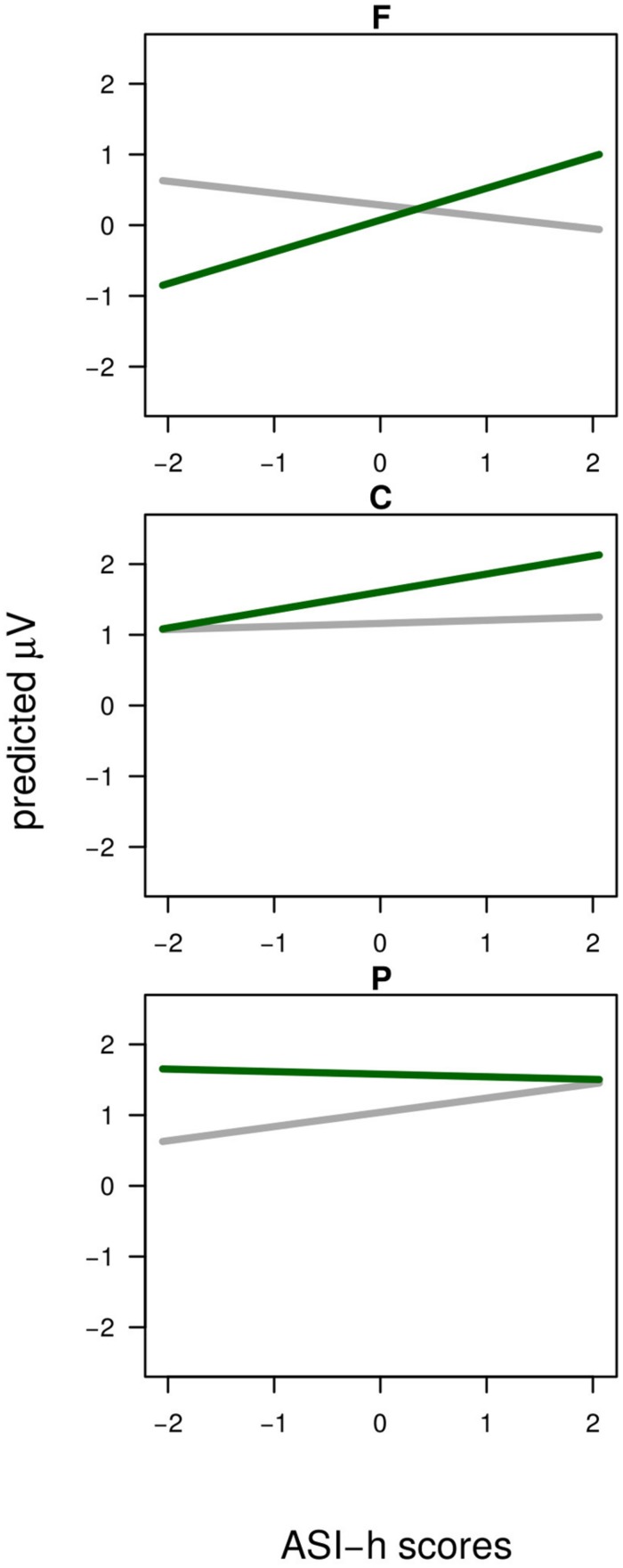
**Stereotypical condition.** The Agreement × Longitude × ASI-h interaction. The *x*-axis is ASI-h scores, and the *y*-axis is amplitude. The solid gray line is the Agreement Match condition and the solid green line is the Agreement Mismatch condition. Top row represents electrodes in Frontal scalp-locations, middle row represents Central scalp-locations and bottom row represents EEG in Parietal scalp-locations.

The analysis revealed two reliable interactions between Agreement and the individual covariates in the Stereotypical condition. One involved Agreement and BSRI-f (**Figure 6**) and was explained by more positive slopes of Mismatch compared to Match condition (β = +0.80, *t* = 2.73) across scalp locations. The crossed slopes suggest that the overall null effect of Agreement is masked by the summation of negative and positive ERP responses to stereotype mismatch. Furthermore, the interaction between Agreement, Longitude, and ASI-h (**Figure 7**) showed slope differences for the agreement effect across levels of longitude: comparing Frontal locations where the slope change was large and positive (β = +0.68) to Parietal electrodes where this change was reduced and negative (β = -0.17) revealed strong differences (βdiff = +0.86, *t* = 5.48) which also emerged in the comparison between Central (β = +0.27), and Parietal electrodes (βdiff = +0.41, *t* = 2.76): less ASI-h scores were associated with a larger Frontal Negativity and larger Posterior Positivity, whereas more ASI-h participants showed a more positive Fronto-Central Positivity.

## Discussion

In the present experiment we investigated the ERP correlates of anaphor processing when the establishment of reference involves the evaluation of gender information. We presented participants with short sentences in which an antecedent was introduced and we recorded the ERPs to the presentation of reflexive pronouns occurring after the verb. We assumed that when a definitional role-noun (*mother*, *father*) is presented, readers access categorical information about the gender of the text character: if the form of the following pronoun is not consistent with the gender of the noun, no available referent can be found thus making the sentence unacceptable. After a stereotypically male or female character is introduced, readers also access information about the gender of the character and create a consistent representation of the discourse. However, upon reading the following pronoun it is possible to find a referent even when stereotype gender and pronoun gender are inconsistent: the counter-stereotypical referent might not be readily available, but because stereotype information is probabilistic and not categorical, it should be possible to search and find it. When nouns have definitional gender and the anaphor cannot be bound to the only available antecedent, a clear P600 effect was found. When a noun conveys gender information through the stereotypical representation associated with it, the ERP correlate of stereotype gender mismatch is biphasic, as showing a negative effect in Frontal Left electrodes and a positive effect in Parietal electrodes. Inspecting individual variability in the ERP response we showed how the biphasic pattern can be explained by the fact that grand averages reflect the summation of two different types of ERP responses: below, we argue that these effects reflect neural activity of Nref and P600 components. Different predictors (BSRI-f, ASI-h, IAT, and Sex) had effects on the ERP response. The different ERP response to gender mismatch in the Definitional and Stereotypical conditions suggest that gender information about stereotypes is not the same information conveyed by definitional gender role-nouns.

The positive part of the effects can be safely interpreted as part of the P600 component. The P600 effect to definitional gender mismatching pronouns confirms previous findings (Osterhout and Mobley, 1995; Osterhout et al., 1997; Hammer et al., 2008; and the literature on morphosyntactic Agreement, see Molinaro et al., 2011). Consistently with grammaticality judgments that fall very low (16%) for definitional gender mismatching pronouns, participants fail to find an appropriate referent for the pronoun. In contrast to what might happen in the processing of free or unbound pronouns (e.g., *he*, *she*) a reflexive pronoun cannot link to a yet unmentioned antecedent, and therefore the observed P600 effect can be taken to reflect a genuine “failure” in linking anaphor and antecedent (consistent with Osterhout and Mobley, 1995).

The biphasic pattern associated with Stereotypical gender mismatching pronouns replicates previous findings only partially: the positive part of the effect is consistent with Osterhout et al. (1997), whereas the overlapping negativity is not. The interpretation of the Left Anterior effect that is visible in the Grand Averages (**Figures 1** and **2**) elicited by stereotype gender mismatching pronouns is not straightforward since different language related ERP components, reflecting the activity of different neural mechanisms, have been described as occurring in Frontal or Left Frontal locations: the focal/morphosyntactic LAN (e.g., Friederici, 2002, 2011), the sustained LAN (e.g., King and Kutas, 1995; Fiebach et al., 2002), and the Nref effect (e.g., Van Berkum et al., 1999). The effect observed here is compatible with modulation of either type of LAN component, but only because the polarity of the effect is negative, and the distribution of the effect is left anterior when looking at the grand averages. In contrast, the timing of the effect and the functional interpretation of the focal LAN do not fit with the effect we observed and the hypothesized undergoing cognitive processes. Focal LANs are usually seen between 300 and 500 ms (i.e., they are not sustained), whereas our effect was sustained (it onsets before 500 ms and is lasts until 900 ms). But the strongest reason to believe that the observed effect is not a LAN effect is that current accounts of the functional meaning of the LAN component (e.g., Friederici, 2011; Molinaro et al., 2011) proposed that LAN should be observed when morphological cues of target and controller in the agreement process are both transparent, and conflicting. In our study, the pronoun’s form provides a transparent morphological gender cue, but for the vast majority of the sentences no gender related morphological cues are provided by English nouns (although a few Definitional nouns, such as *actress* or *mistress* convey morphologically transparent – and female – cues). Therefore, one crucial condition for eliciting “morphosyntactic” LAN effects is not met. And even if we assumed that LAN is triggered by disconfirmed syntactic predictions (as is also proposed in Molinaro et al., 2011) it is clear that syntactically driven expectations should be much stronger when gender information is categorical. On this hypothesis, we should have observed a more negative LAN in the definitional condition. In fact, it was more pronounced in the stereotypical condition. Therefore the negative effect observed here cannot be interpreted as a focal LAN.

Alternatively, the effect may look more similar to the “sustained” LAN which has been found in the processing of long distance syntactic dependencies (e.g., King and Kutas, 1995; Fiebach et al., 2002) and has been associated with working memory costs for holding open gaps in the syntactic representation of the sentence. But this functional interpretation also does not fit, because if any gap has been opened (at the Verb) it should be “filled” when processing the reflexive. Our preferred interpretation is, therefore, that the Left Anterior effect is an Nref effect (Van Berkum et al., 1999, 2003, 2007; Nieuwland and Van Berkum, 2006; Nieuwland, 2014). Nref effects have been reported in cases in which two or more antecedents are equally plausible referents for an anaphor (Nieuwland and Van Berkum, 2006; Nieuwland et al., 2007), or when a mismatch occurs between the only available antecedent and an unbounded pronoun, that can be linked to an as yet unmentioned, unknown referent associated with the discourse (Nieuwland, 2014). Based on these findings, the Nref effect has been taken to reflect the search for additional information to link anaphor and antecedent. In the present experiment we manipulated the relation between anaphor and antecedent and it is likely that, when processing Stereotypical gender mismatching pronouns, participants might need to look for additional information to realize that antecedent and pronoun are coreferential, even though a *mechanic* is more often *male* than *female*. Stereotypical gender information is a probabilistic bias that guides the assignment of a male/female feature to a role-noun, but does not determine the antecedent gender categorically. Consistently with this idea, the acceptability ratings for stereotype gender mismatch passages are very high (89%) showing that (at least at the end of the sentence) pronouns and antecedent are judged as coreferential, although the corresponding sentences were still perceived as less well formed than stereotype matching sentences (94%). The distribution of the effect we observed may seem at odds with the canonical distribution of the Nref effect that tends to be bilateral, but a few examples of more left lateralized Nrefs have been reported (Experiments 1 and 2 in Nieuwland, 2014; Figure 1 in Nieuwland and Van Berkum, 2008). Moreover, although the Grand Averages show a frontal left distribution, the effects of the covariates (see below) often interacted with the agreement pattern and the Longitudinal rather than the Mediality dimension. Therefore we believe that the particular distribution of the effect is due to the summation of two types of “late” ERP responses: a broad anterior Nref and a posterior P600.

Interesting insights derive from our investigation of individual differences. Differences between male and female participants were reported by Osterhout et al. (1997): gender violations (both stereotypical and definitional) elicited larger P600 responses for female subjects than for male subjects. We also found differences between Male and Female participants but they emerged only in relation to individual covariates and, somewhat unexpectedly, following Definitional rather than Stereotypical role nouns: an increase in IAT scores was associated with larger P600 effects to the processing of definitional gender mismatching pronouns for male participants only; furthermore, an increase in BSRI-f scores for male participants was related to smaller positive effects to definitional gender mismatch in both Frontal Central and Parietal electrodes, whereas female participants showed a similar pattern only in Parietal and Central electrodes, while in Frontal electrodes the effect was reversed, with larger Frontal positive effect for higher BSRI-f scores. The lack of strong asymmetries between Female and Male participants could be due to the use of the individual covariates that might have captured the EEG variance better then a dichotomous variable such as participants’ sex. To review the effects of covariates that affected the EEG amplitude independently of participant sex, we notice that BSRI-f and ASI-h were the most relevant. BSRI-f appeared to modulate agreement following both Definitional and Stereotypical rolenouns: when role-nouns gender information was stereotypical, participants that described themselves as less feminine showed also a larger negative response to mismatching pronouns, but when the gender was semantically defined the low BSRI-f participants showed larger P600 effects in Central and Parietal electrodes; on the other hand, male participants with more “Feminine Traits” showed a reduced size of the P600 effect across all scalp sites but an increased Frontal P600 if participants were female.

We believe that the finding that both BSRI-f and ASI-h were associated with the size of the Negative effect in Frontal electrodes (mainly with Stereotypical role nouns, but to some extent also with Definitional role nouns), and that these interactions did not involve differences across levels of Mediality, support the idea that the observed negativity is not strongly lateralized and thus the ERP pattern can be described as the temporal overlap of a frontally distributed Nref with the P600 effect in Parietal electrodes (that in the Grand Averages shows a more left-lateralized distribution).

Differences between the present study and that of Osterhout et al. (1997) may partly explain the differences between their results and ours. Was the British Brightonian sample more liberal than the American Seattle sample in 1997? Do differences in stereotype bias exist between countries (Misersky et al., 2013)? Is today’s society less biased than 15 years ago? And if so, was it the efforts of governments that helped to reduce the gender gap? Clearly these questions cannot be easily answered from a psycholinguistic perspective, which instead suggests alternative hypotheses. One is that the linguistic materials were slightly more biased in Osterhout et al. (1997), because of the use of adjectives or other modifiers, which might have induced stronger commitment to probabilistic gender information, either because of further gender biasing in the modifiers themselves or because the presence of modifying information encouraged a more highly specified representation of the person. Another possibility is that because Osterhout et al. (1997) had lower spatial density in the EEG recording (13 electrodes in total), they might have missed the effect over frontal left electrodes revealing the biphasic pattern. In both Osterhout and Mobley (1995) and Osterhout et al. (1997) some hints of a frontal negativity can be seen by inspecting their figures. In the penultimate paragraph of their study, Osterhout et al. (1997, p. 282) acknowledge the unexpected nature of their findings: “Anomalies involving social categories that are not marked in the grammar (e.g., race) should not elicit the P600 effect but might elicit the N400 effect associated with semantic/pragmatic aspects of language”. On the basis of the present results we believe that their idea that social categories should not elicit the same response as the response for grammatically encoded linguistic features was correct but the prediction of a N400 component effect was disconfirmed by their and the present study’s results: stereotype gender mismatch did not elicit an N400 but rather a Nref effect as an index of inferencing about the most suitable referent of the discourse (Van Berkum, 2009).

If the mechanisms underlying the P600 in reflexive pronouns processing can be taken to reflect a failure to link the anaphor with the antecedent, when processing sentences with stereotypical gender role nouns, the P600 effect suggests that participants behave as if sometimes the link between gender inconsistent pronouns and antecedents cannot be established, whereas participants with lower scores in the BSRI-f or ASI-h that show an Nref effect suggest that less Feminine or less explicitly sexist participants may have actively searched for an appropriate although less likely antecedent. The modulation of the size of the Nref and P600 components may be linked to the strength of the stereotype bias that participants use to create the gendered representation of the text characters. The study of individual variation in the ERP response was fruitful because it allowed us to distinguish two ways in which co-reference can be evaluated when gender information is not categorical. One way is to use stereotype information as a categorical feature, perceiving the mismatch as an agreement violation (at least initially – by the end of each sentence most ratings turn out to be “acceptable”). The other way is to consider it as indicating a case of possible referential ambiguity, which requires additional processing effort to search for the possible although less likely referent. We believe that the complex pattern of interactions between individual measures of sexism and the way the anaphoric relation is evaluated is an interesting finding, because it suggests that language processing depends on participants’ characteristics that are unrelated to language competence. However it is not straightforward to explain the observed relation between personality traits and anaphor resolution. For instance, BSRI-f traits are termed “expressive” in the literature critiquing the BSRI (Payne, 1985; Choi and Fuqua, 2003). Participants who described themselves as not having the traits regarded as desirable when attributed to women in 1974 (less “Affectionate”, less “Cheerful”, less “Childlike”, less “Compassionate”, less “Does not use harsh language”) appear to have been more actively engaged in trying to resolve the loose agreement between anaphor and antecedent when it involved stereotypical representations. Those who, on the other hand, had a more “expressive” self-representation, were either more sensitive to stereotypical information, or less prone to search for a counter-stereotypical representation of a role-noun.

Overall, the present study suggests that cognition can be better described when accounting for individual variation and, importantly, that variation in a linguistic task can be predicted also on the basis of personality factors that are largely independent of linguistic competence: likewise, researchers in the framework of cognitive psychology may benefit from investigating the effect of non-domain-specific factors that may not seem obviously relevant. The reported evidence is consistent with the view that language comprehension is influenced by the larger (non-linguistic) context of individuals’ experience and personal beliefs, which likely plays a role in generating the mental representation of the text, of a communicative interaction, or more generally of the situation model.

## Conflict of Interest Statement

The authors declare that the research was conducted in the absence of any commercial or financial relationships that could be construed as a potential conflict of interest. The reviewer Stefanie Nickels and handling Editor declared their shared affiliation, and the handling Editor states that the process nevertheless met the standards of a fair and objective review.

## Footnotes

1 http://psy.ck.sissa.it

2 http://faculty.washington.edu/agg/iat_materials.htm

3 www.neurobs.com

4 http://cran.r-project.org
